# NASTRA: accurate analysis of short tandem repeat markers by nanopore sequencing with repeat-structure-aware algorithm

**DOI:** 10.1093/bib/bbae472

**Published:** 2024-09-25

**Authors:** Zilin Ren, Jiarong Zhang, Yixiang Zhang, Tingting Yang, Pingping Sun, Jiguo Xue, Xiaochen Bo, Bo Zhou, Jiangwei Yan, Ming Ni

**Affiliations:** Changchun Veterinary Research Institute, Chinese Academy of Agricultural Sciences, State Key Laboratory of Pathogen and Biosecurity, Key Laboratory of Jilin Province for Zoonosis Prevention and Control, No. 573 Yujinxiang Street, Jingyue District, Changchun 130012, China; School of Information Science and Technology, Northeast Normal University, No. 2555 Jingyue Street, Jingyue District, Changchun 130117, China; School of Forensic Medicine, Shanxi Medical University, No. 55 Wenhua Street, Yuci District, Taiyuan 030001, China; Changchun Veterinary Research Institute, Chinese Academy of Agricultural Sciences, State Key Laboratory of Pathogen and Biosecurity, Key Laboratory of Jilin Province for Zoonosis Prevention and Control, No. 573 Yujinxiang Street, Jingyue District, Changchun 130012, China; School of Information Science and Technology, Northeast Normal University, No. 2555 Jingyue Street, Jingyue District, Changchun 130117, China; School of Forensic Medicine, Shanxi Medical University, No. 55 Wenhua Street, Yuci District, Taiyuan 030001, China; School of Information Science and Technology, Northeast Normal University, No. 2555 Jingyue Street, Jingyue District, Changchun 130117, China; Advanced & Interdisciplinary Biotechnology, Academy of Military Medical Sciences, No. 27 Taiping Road, Haidian District, Beijing 100850, China; Advanced & Interdisciplinary Biotechnology, Academy of Military Medical Sciences, No. 27 Taiping Road, Haidian District, Beijing 100850, China; Changchun Veterinary Research Institute, Chinese Academy of Agricultural Sciences, State Key Laboratory of Pathogen and Biosecurity, Key Laboratory of Jilin Province for Zoonosis Prevention and Control, No. 573 Yujinxiang Street, Jingyue District, Changchun 130012, China; School of Forensic Medicine, Shanxi Medical University, No. 55 Wenhua Street, Yuci District, Taiyuan 030001, China; Advanced & Interdisciplinary Biotechnology, Academy of Military Medical Sciences, No. 27 Taiping Road, Haidian District, Beijing 100850, China

**Keywords:** nanopore sequencing, short tandem repeats, cell line authentication, accurate analysis method

## Abstract

Short-tandem repeats (STRs) are the type of genetic markers extensively utilized in biomedical and forensic applications. Due to sequencing noise in nanopore sequencing, accurate analysis methods are lacking. We developed NASTRA, an innovative tool for Nanopore Autosomal Short Tandem Repeat Analysis, which overcomes traditional database-based methods’ limitations and provides a precise germline analysis of STR genetic markers without the need for allele sequence reference. Demonstrating high accuracy in cell line authentication testing and paternity testing, NASTRA significantly surpasses existing methods in both speed and accuracy. This advancement makes it a promising solution for rapid cell line authentication and kinship testing, highlighting the potential of nanopore sequencing for in-field applications.

## Introduction

Short tandem repeats (STRs) in the human genome are specific deoxyribonucleic acid (DNA) loci that can have a high degree of polymorphisms in the number and forms of repeat motifs among individuals. This feature makes STRs a kind of genetic markers well-suited for individual identification [1, 2], cell line authentication [3, 4], and relationship inferring [5–7]. STR genetic markers are usually selected considering high heterozygosity, regular repeat units, distinguishable alleles, and robust polymerase chain reaction amplification. The Short Tandem Repeat DNA Internet Database (STRBase) maintained by the National Institute of Standards and Technology [8] includes >70 human STR genetic markers, with numbers of known per-locus alleles ranging from 19 (D10S1248) to 114 (FGA locus). These STRs have been utilized for decades by laboratories worldwide and have formed large-scale human DNA databases for public security purposes in many countries. In biomedical research, STR profiling is used for cell-line authentication, which plays an important role in ensuring scientific reproducibility and preventing misidentification as well as cross-contamination of cell lines [9, 10]. The International Cell Line Authentication Committee (https://iclac.org) integrates many online databases and search tools, such as the Cellosaurus knowledge resource [11], DSMZ STR profile database [12], CLIMA database [13], and CLASTR [14].

The traditional approach to STR profiling relies on capillary electrophoresis (CE)-based length analysis. Massively parallel sequencing (MPS) has also been validated for use in STR profiling, offering the capability to analyze a broader array of STRs in a single run compared to the CE method. However, both CE and MPS systems for STR profiling have their limitations: they are immobile, expensive, and require specific environmental conditions, such as stable temperature and absence of vibration [15, 16]. Consequently, these instruments are typically housed in well-equipped laboratories or specialized facilities. This necessitates the transportation of samples for analysis, often leading to delays of several days before obtaining STR typing reports [17].

Nanopore sequencing offers an economical and flexible alternative for STR profiling. Devices like the MinION Mk1B/Mk1c (Oxford Nanopore Technologies, Oxford, United Kingdom) and QNome-3841 (Qitan Technology, Chengdu, China) stand out for their exceptional portability and lower cost compared to CE and MPS systems. These portable nanopore sequencing devices not only produce longer reads than Sanger sequencing and NGS systems but also yield data at the Gbp level in a single run. Nanopore sequencing has found diverse applications, including on-site genomic surveillance of pathogens [18, 19] and biodiversity studies [20, 21]. Zaaijer et al. [17] conducted a pilot study using the MinION device for rapid whole genome sequencing to authenticate human cell lines in biological research.

Despite these advantages, nanopore sequencing is noisier than CE and MPS, which is challenging for the accurate analysis of the STR markers [15, 22–24]. For the detection of kilobase-length STRs in repeat expansion disorders, tools like repeatHMM [25], STRique [26], and DeepRepeat [27] have been developed. Compared to detecting abnormally long expansions, conducting accurate genotyping calls for cell line identification and forensic applications presents greater challenges. The alleles of these STR genetic markers typically range from 100 to 300 nucleotides (nt) in length and are composed of 3- to 5-nt repeat units. The genotypes of these STR markers are determined by the precise count of their repeat units. We previously reported that tools designed for detecting repeat expansions, like repeatHMM [25], are not suitable for STR genetic markers. In contrast, employing the classic Smith–Waterman algorithm for re-alignment significantly enhances the accuracy of STR typing, as noted in our earlier findings [28]. Hall et al. [24], Tytgat et al. [15], and recently Lang et al. [29] have employed a straightforward strategy. Their approach involves aligning all available sequences of STR alleles and selecting the alleles with the most supporting reads as the genotypes. However, this method relies on allele databases, which can lead to typing errors for carriers of unknown alleles. In the meantime, owing to the inherent noise in nanopore sequencing, there is a heightened risk of errors when using database-based strategies for alleles with highly similar nucleotide sequences.

To address the precision challenges in STR typing using nanopore sequencing, we introduced Nanopore Autosomal Short Tandem Repeat Analysis (NASTRA), an algorithm specifically developed for accurate STR analysis without reliance on reference database alignment. It is particularly suited for cell line authentication, as well as individual and kinship identification. NASTRA utilizes read clustering to reduce the impact of subtle sequencing errors and features a recursive algorithm for inferring the repeat structure of alleles based solely on known repeat motifs. Our evaluation, encompassing various multiplex systems and flow cells, demonstrated NASTRA’s effectiveness in genotyping 76 individual samples across 27 autosomal STR markers. Additionally, genotyping calls on eight standard cell lines resulted in high accuracy. We further validated NASTRA in individual identification and paternity testing within a four-member family and two unrelated individuals. The results demonstrated that NASTRA could accurately confirm all individual identifications and paternity tests within sequencing durations of 12 and 18 minutes, respectively. Overall, NASTRA exhibits impressive performance in both accuracy and speed.

## Methods

### Study overview

**Figure 1:**
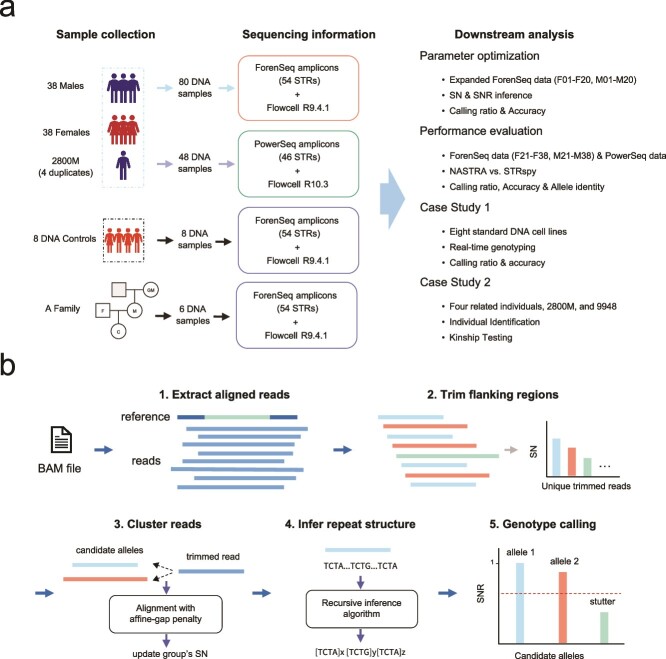
The overview of the study design. (a) We carried out nanopore sequencing on a collection of 76 individual samples, along with duplicated 2800Ms, using various amplification kits and flow cells. This was done for both parameter optimization and performance evaluation. Subsequently, we performed two case studies, one involving cell line profiling and the other focused on paternity testing. (b) The framework of NASTRA. NASTRA consists of five steps to perform genotyping calls.

A total of 76 blood samples were obtained from unrelated, anonymized Han Chinese volunteers, comprising 38 females (ID: F01–F38) and 38 males (ID: M01–M38). Of these, 30 samples (F01–F15 and M01–M15) were sourced from our previous study [28], while the remaining 46 samples (F16–F38 and M16–M38) were newly acquired. Besides these, we also gathered eight control DNA samples. These included three forensic DNA controls (2800 M from Promega; 9947A and 9948 from OriGene), along with five cell line standards (NA12878, NA24143, NA24149, NA24694, and NA24695 from the Coriell Institute). Additionally, for a case study on paternity testing, we included a family of four: a grandmother, mother, father, and child (Fig. 1a). The details of dataset can be found in the Supplementary material.

NASTRA requires three inputs: (i) the alignments of nanopore reads to the human reference genome; (ii) the reference genomic positions of STR markers; and (iii) the repeat motifs stored in the STR fact sheets. NASTRA’s pipeline consists of two primary components: read clustering and repeat structure inference. These two components are designed to mitigate the potential impact of subtle sequencing errors and make accurate genotyping calls without the allele reference database (Fig. 1b). In step 1, NASTRA extracts all reads spanning the specified STR loci from the BAM file. In step 2, to obtain the STR core region, NASTRA trims both prefix and suffix flanking regions using local pairwise alignment with an affine-gap penalty (Supplementary data). In detail, NASTRA will retain an additional three bases adjacent to the core region on both sides. In step 3, NASTRA clusters all trimmed reads using global pairwise alignment with an affine-gap penalty, where reads that are identical or 1-bp mismatch are considered as one group and the number of reads in a group will be considered as the supporting read number (SN). The top three sequences with the highest SN are selected as candidate alleles. In step 4, we have developed a recursive algorithm that infers the repeat structure of each allele sequence based on repeat motifs. This approach not only facilitates the rapid acquisition of STR genotypes but also assists in the quick identification of single nucleotide variants in genotypes. Ultimately, NASTRA carries out STR genotyping by utilizing the SN and the supporting read number ratio (SNR), where SNR is calculated as the ratio of an allele’s SN to the SN of the major allele. Drawing inspiration from the quality control mechanism used in the ForenSeq Universal Analysis Software (UAS), NASTRA incorporates similar rules. These rules are designed to filter out unreliable genotyping results characterized by low coverage and stutters, thereby enabling more accurate genotyping calls.

### Repeat structure inference and genotyping calls

**Figure 2:**
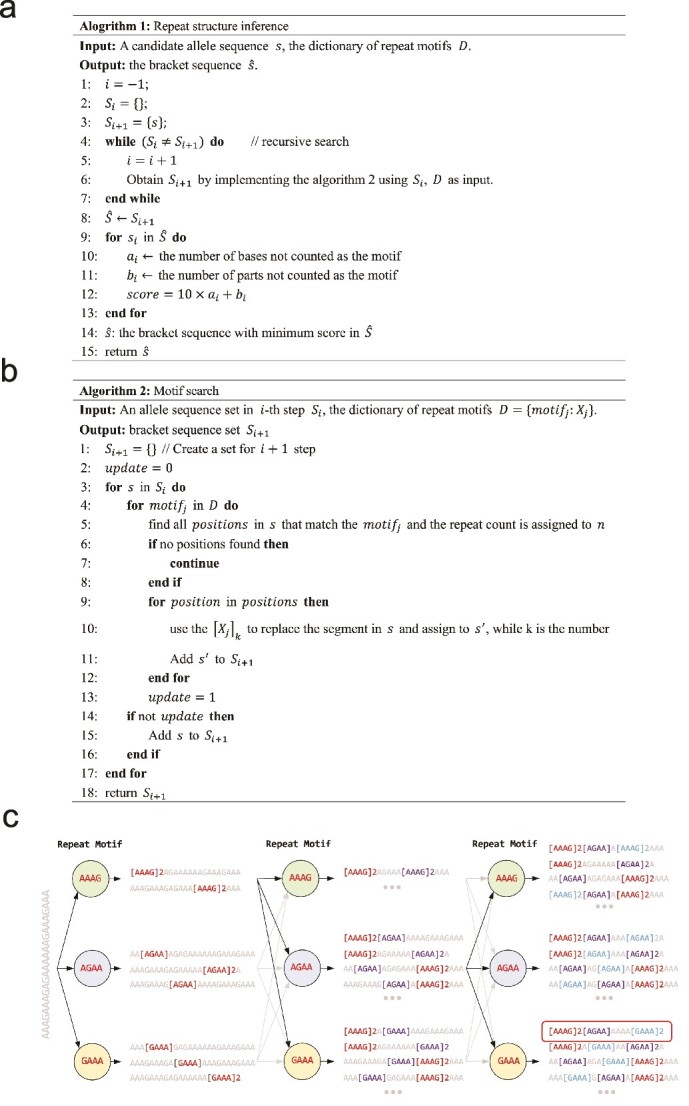
The recursive algorithm for repeat structure inference. (a) Pseudocode for the repeat structure inference. This algorithm utilizes the motif search method described in (b), employing recursion to achieve the structure inference. (b) Pseudocode for the motif search algorithm. (c) This schematic illustrates the process of inferring repeat structure. The sequence on the left represents the candidate allele. In each step, motifs, AAAG, AGAA, and GAAA, are searched one by one. As the process progresses, identified fragments in each step are transformed into bracket sequences.

Repeat motifs for each STR were collected from the STR fact sheet of STRBase [8]. The algorithm is designed to recursively search for sequence fragments that match these repeat motifs. To elucidate the process, we provided pseudocode (Fig. 2a and b) and an example (Fig. 2c). NASTRA begins by searching for each motif (AAAG, AGAA, and GAAA) within a given sequence (depicted in full gray). After finding these motifs, it identifies eight unique fragments and transforms them into eight distinctively-marked sequences. The algorithm then repeats the search for each marked sequence. This iterative process is carried out until no new fragments are found, or the iteration count exceeds 50. Finally, NASTRA calculates the count of non-motif bases and parts for each marked sequence. The sequence with the lowest score (defined in Fig. 2a) is then determined to be the most plausible inferred structure (highlighted in a red box). For each structured allele, NASTRA sums up the counts of repeat motifs to infer its genotype. However, the presence of certain single nucleotide polymorphisms (SNPs) in the adjacent and repeat regions [30] can lead to challenges in making accurate genotyping calls. To address this, locus-specific adjustments have been incorporated into NASTRA. Detailed information about these adjustments is available in the Supplementary method.

NASTRA determines the STR’s genotype based on the alleles’ SNs and SNRs. In more detail, if the highest SN among the alleles is <10, NASTRA will categorize the result as “Interpretation”, indicating an interpretation failure due to inadequate coverage. Meantime, NASTRA assesses whether the STR genotype is heterozygous or homozygous based on the SNR of the allele with the second-largest SN. A heterozygous result is declared if the SNR of the minor allele surpasses a predefined threshold. Conversely, if the SN of the minor allele falls below this threshold, but the major allele’s SN exceeds it, the result is labeled as “imbalance”. For the optimal selection of SN and SNR thresholds, we have included a configuration file for users.

## Results

### Parameter optimization using downsampled data

To infer the optimal parameters for NASTRA, we split the samples into two sets: (i) training set, comprising 20 males and 20 females, and (ii) test set, consisting of 18 males, 18 females, and 4 replicates of 2800 M samples. Considering the limited size of the training set and the insensitivity of the deep sequencing depths toward the thresholds, we expand the training and test sets using the tool NanoTime. To assess the performance of NASTRA, we used the benchmark set built by the MiSeq FGx system, where the typing results with “imbalanced”, “allele count”, “stutter”, and/or “interpretation threshold” were excluded (Supplementary method).

First, we used NASTRA to genotype alleles for 12 960 STRs on 27 autosomal STR loci across 480 samples from the train set. Then, we used various SN values (ranging from 0 to 50 in increments of 5) as well as SNR values (ranging from 0 to 1 in increments of 0.01) to accomplish the genotyping calls for these STRs. The genotyping results with “Interpretation” and “Imbalance” were removed. Finally, we compared the NASTRA’s genotyping results to the benchmark set, and the results were classified into five categories: (i) exact match: correct genotyping; (ii) incomplete match: only the incomplete repeat unit is different from the correct genotyping; (iii) one match: one of the alleles is genotyped correctly; (iv) incomplete one match: one of the alleles is correctly genotyped when the incomplete repeat unit is ignored; and (v) mismatch: incorrect genotyping. In our study, we defined accuracy as the proportion of all samples that were correctly typed at the STR locus. If all samples were correctly typed, the accuracy would be 100%. For calling ratio, it is defined as the proportion of all samples that could be successfully typed at the STR locus using NASTRA.

**Figure 3:**
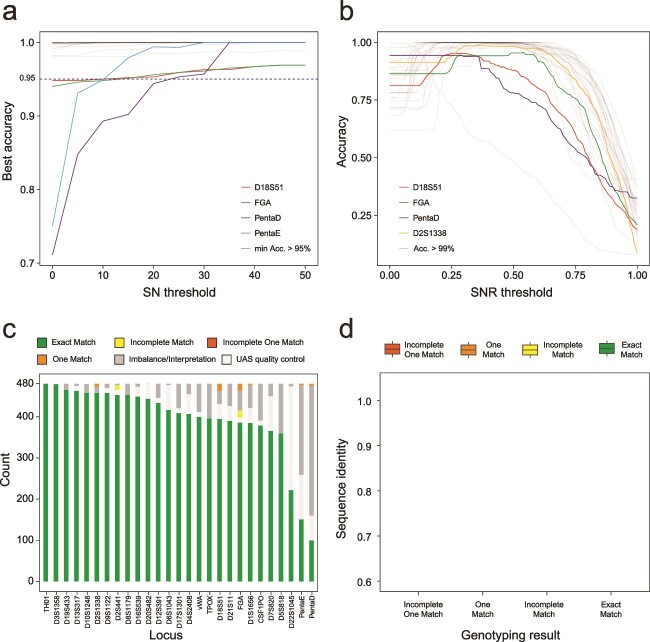
Parameter optimization using downsampled data. (a) NASTRA’s highest accuracy on each locus with a fixed SN. Each line represents a locus, and the gray lines are loci whose typing accuracy is always greater than 95%. (b) The genotype calling accuracy of NASTRA on each locus at various SNR, with the SN fixed at 25. Each line represents a locus, and the gray lines are loci whose peak typing accuracy surpasses 99%. (c) The statistical diagram presents the genotyping results from downsampled data. (d) The blast identity of allele sequences predicted by NASTRA with the benchmark sequences. In (c) and (d) dark green indicates an exact match, light green signifies an incomplete match, yellow for one match, orange for an incomplete one match, and red denotes a mismatch.

For each SN value, we calculated the maximum genotyping accuracy for each autosomal STR locus on the expanded training set by varying SNRs. The result showed that, when SN reaches or exceeds 25, the maximum genotyping accuracy for all STR loci can exceed 95% (Fig. 3a). After having determined the SN value of 25, we calculated the genotyping accuracy for each autosomal STR across different SNR values. The SNR threshold is defined as the median value within the range of SNR values corresponding to the peak genotyping accuracy (Fig. 3b). After having determined the threshold SN and SNR, NASTRA was used to perform STR genotype calling on the expanded test set.

In summary, 2758 of 12 960 genotype calls failed to pass the quality control, including 936 calls in the benchmark set and 1822 calls by NASTRA, which is mainly caused by insufficient calls with short sequencing duration (Supplementary Fig. S1). The rest 10 202 of 12 960 (78.72%) STRs on 480 expanded test sets were genotyped successfully, consisting of 9817 (96.23%) exact matches, 45 (0.44%) incomplete matches, 313 (3.07%) one matches, 12 (0.12%) incomplete one matches, and 15 (0.15%) mismatches. Figure 3c depicts the accuracy of each STR across the 480 samples from the test set. Out of the 27 loci, 13 loci had a genotyping accuracy of 100% (exact match), and 7 loci had an accuracy greater than 95% (Supplementary Table S1). Additionally, we employed pairwise alignment to investigate sequence identity for each allele (Fig. 3d).

### Evaluation of NASTRA on ForenSeq amplicons with R9.4.1 flow cells

**Figure 4:**
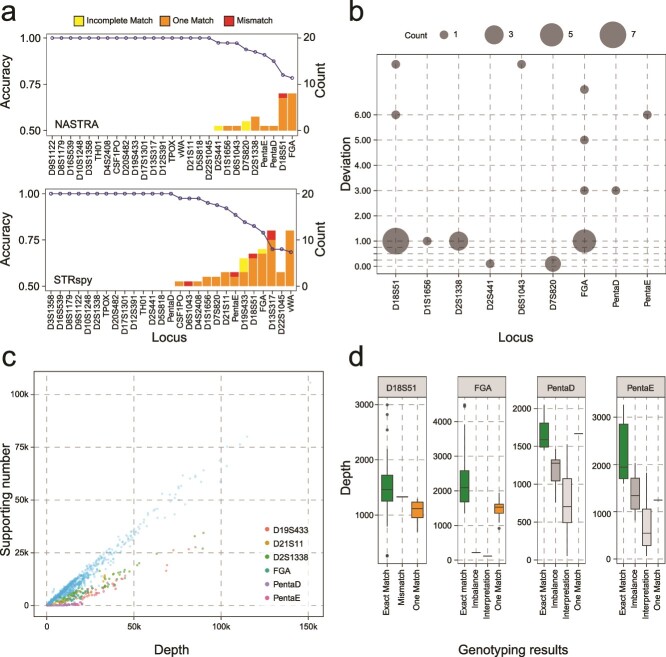
Performance evaluation on ForenSeq data with R9.4.1 flow cells. (a) Accuracy comparison between NASTRA and STRspy. (b) Genotype deviations from baseline as calculated by NASTRA. For heterozygous genotypes, the sum of the deviation for both alleles were calculated. (c) The correlation of SN and sequencing depth. (d) Distribution of sequencing depth across various genotyping results at four loci.

We assessed the performance of NASTRA on ForenSeq amplicon sequencing data with a sequencing duration of 24 hours, comparing it to the database-based method, STRspy [24]. STRspy is an STR profiling tool tailored for long-read sequencing, which aligns reads to the allele reference database. STRspy provides a customized database that contains 638 allele sequences of 22 autosomal STRs. We then built a database of allele reference for the rest 5 STR loci (D17S1301, D20S482, D4S2408, D9S1122, and D6S1043) using the allele information provided by the STRBase (https://strbase-b.nist.gov). We first used the calling ratio, the proportion of samples successfully genotyped at a locus, to assess both tools. There are 27 loci exhibiting a calling ratio of 1 using STRspy. Regarding NASTRA, the calling ratios for 25 out of the 27 loci range from 94.4% to 100%, as illustrated in Supplementary Fig. S2. For genotyping accuracy, NASTRA genotyped accurately 18 loci across all samples, while STRspy successfully genotyped 14 loci correctly (Fig. 4a, Supplementary Table S2). Figure 4b shows that most deviations between true and imputed alleles are 1, which may be due to the SNR threshold setting for D18S51 and FGA. While using STRspy, the absence of some specific allele information influenced the genotyping accuracy. For instance, the FGA’s allele 24.2 of sample F25 is [GGAA]2_GGAG_AA_[AAAG]16_[AGAA]1_AAAA_ [GAAA]3, while the allele in the reference database is in the [GGAA]2_GGAG_[AAAG]17_AA_AAAA_[GAAA]3. Thus, STRspy predicted the allele as 24, leading to an incomplete match with the wrong repeat structure. This highlights the importance of a comprehensive and highly reliable reference database for database-based methods to achieve precise genotyping.

To investigate the causes of incorrect genotyping results, we analyzed the relationship between SN and sequencing depth. Figure 4c indicates that the loci with incorrect genotyping have lower SNs than those with correct genotyping when the sequence depth is close. This phenomenon may be attributed to the presence of homopolymers containing adenine (A) and guanine (G), as observed in loci such as FGA, PentaD, and PentaE. A possible solution is to increase the sequencing depth for these loci (Fig. 4d).

### Evaluation of NASTRA on PowerSeq amplicons with R10.3 flow cells

To assess the performance of NASTRA on different amplification systems and flow cells, we conducted a sequencing experiment on 46 samples using Promega’s PowerSeq 46GY kit and R10.3 sequencing flow cells. The PowerSeq kit is a co-amplification system consisting of 22 autosomal STR loci and 23 Y-STR loci. The ONT R10.3 flow cell represents a new version featuring a new pore design, resulting in enhanced read accuracy and quality.

**Figure 5:**
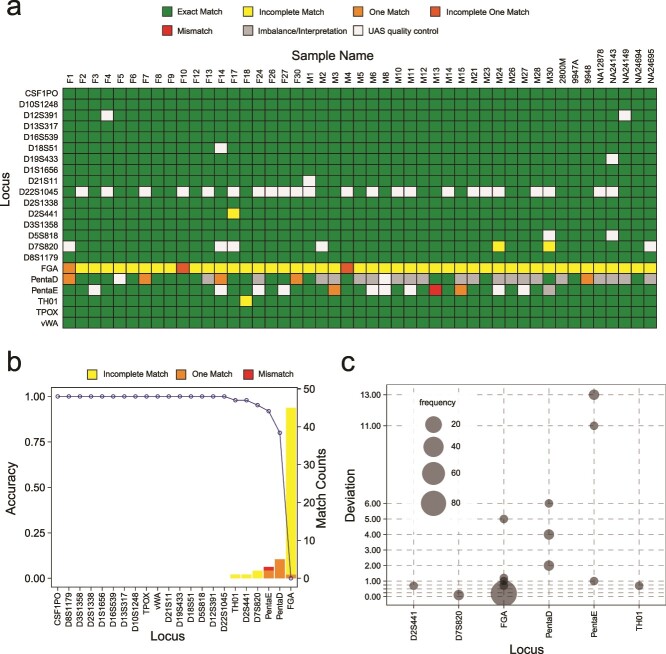
Performance evaluation on PowerSeq data with R10.3 flow cells. (a) Overview of genotyping results on 22 autosomal STRs across 46 samples. (b) Accuracy comparison between NASTRA and STRspy. (c) Genotype deviations from baseline as calculated by NASTRA. For heterozygous genotypes, the sum of the deviation for both alleles were calculated.

Using the optimal parameters inferred on the ForenSeq data with R9.4.1 flow cells, NASTRA showed the robustness of accuracy on the PowerSeq data (Fig. 5a). For NASTRA, among the 22 autosomal STR loci, 20 STR loci exhibited a calling ratio of 1 (Supplementary Fig. S3), with only the remaining 2 loci, PentaE (94.7%) and PentaD (55.6%). NASTRA demonstrated superior performance over STRspy, with 16 out of 22 loci correctly typed compared to 12 (Fig. 5b and Supplementary Fig. S4). Specifically, NASTRA achieved an accuracy of >95% in 19 loci, compared to STRspy’s 15 loci (Supplementary Table S3). Meanwhile, STRspy achieved an accuracy between 13% and 93.5% in the remaining 7 loci. In Fig. 5a and c, we found that almost all FGA typing results were marked as incomplete matches, and NASTRA identified incomplete units, while the true typing did not contain incomplete units. This indicates that there may be sequencing noise in homopolymer region, causing [AGAA]n AAAA [GAAA]m to become [AGAA]n AA [GAAA]m.

### Case study: typing of eight human cell lines

**Figure 6:**
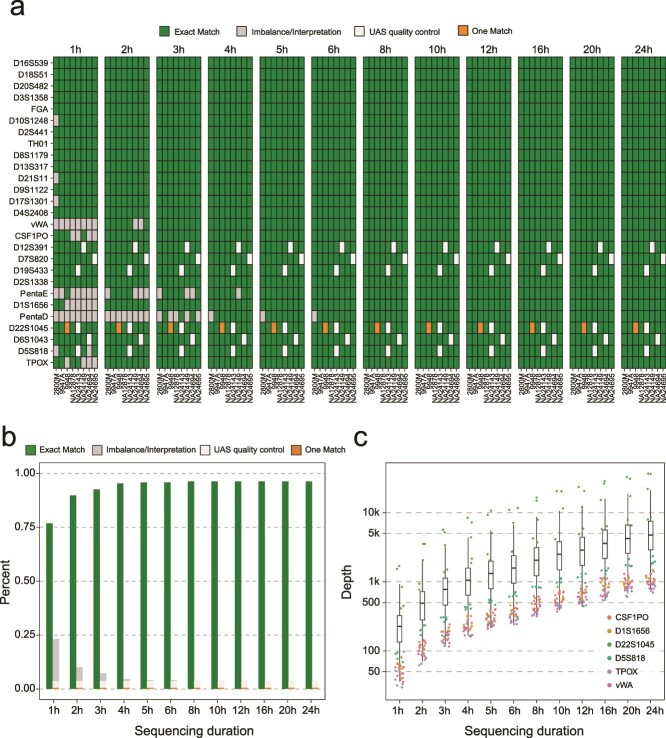
Performance evaluation of profiling eight standard cell lines. (a) Overview of genotyping results of 8 standard DNA samples across 27 autosomal STRs with different sequencing durations using an R9.4.1 flow cell. Gray represents unreliable results, and white represents genotypes that did not pass UAS quality control in the benchmark construction. (b) The statistical diagram presents the proportion of genotyping results at various sequencing durations. (c) Distribution of sequencing depth at 27 autosomal STR loci over different sequencing times. D22S1045 shows the highest sequencing depth, whereas CSF1PO and four other loci consistently show low sequencing depths.

We evaluated NASTRA for cell line authentication by profiling the STRs in eight individual cell lines. For this, we processed DNA samples using the ForenSeq Signature kit and then performed sequencing on a MinION platform with an R9.4.1 flow cell. The genotyping results with different sequencing durations are shown in Fig. 6a. Excluding unreliable genotyping calls and those that failed UAS quality control in the benchmark, NASTRA accurately genotyped all samples except D22S1045 of 9948 (labeled as one match). When the sequencing time extended to four hours, the CODIS core loci (13 autosomal STRs) across all individuals were genotyped correctly, demonstrating NASTRA’s sufficiency for individual identification and cell line authentication. Notably, our analysis solely concentrated on autosomal STRs, which means a portion of the sequencing throughput of other markers (24 Y-STRs, 7 X-STRs, and 94 identity SNPs) was underutilized. This suggests that the sequencing duration required for cell line authentication could be shorter. We found that the unreliable genotyping results were mainly caused by the sequencing depth, as shown in Fig. 6b. When the sequencing depth for each locus exceeds 500, the number of unreliable genotyping results would be very few (Fig. 6c). This would provide a certain reference for the design of the multiplex panels tailored for nanopore sequencing.

### Case study: paternity testing on a family of four individuals

**Figure 7:**
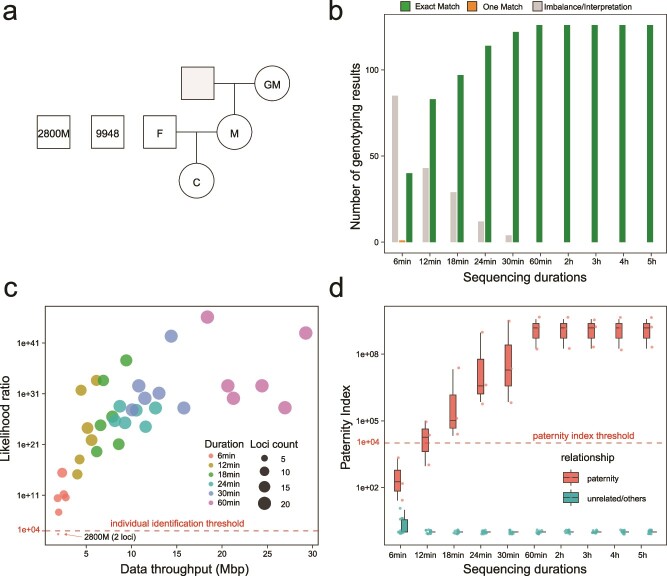
A case study of using NASTRA to perform individual identification and paternity testing. (a) Sample and family information. (b) Counts for different genotyping results with varying sequencing durations. (c) Likelihood ratios for individual identification. The X-axis represents the sequencing throughput of each individual. The size of the dots represents the number of loci successfully and correctly genotyped. The colors represent for sequencing duration. (d) Distribution of paternity index scores for parent–child paternity testing with various sequencing durations.

To further evaluate NASTRA, we carried out a paternity test on a family of four with two unrelated DNA samples, 2800 M and 9948, as controls (see Fig. 7a). The benchmark for the test was established using CE and the commercial PowerPlex 21 kit. For nanopore sequencing, we utilized the ForenSeq Signature kit to obtain amplicons and employed an R10.3 flow cell for the sequencing. For the purpose of objective comparison, we only used 21 loci in the PowerPlex 21 kit to determine genetic relationships. We first summarized the sequencing throughput at different durations (Supplementary Table S4). On the individual level, the MinION yielded sequencing data ranging from 1.84 Mbp (M, 6 minutes) to 95.50 Mbp (F, 5 hours). We then summarized NASTRA’s genotyping results on 21 STR loci among 6 individuals (126 genotyping results, Fig. 7b). When the sequencing duration was 6 minutes, the majority of genotyping results did not meet quality control standards, primarily due to inadequate sequencing yields. When the sequencing duration reached 60 minutes, all STR loci were correctly genotyped. To explore the sequencing duration required for individual identification and paternity testing, we calculated the likelihood ratio for individual identification and the paternity index for individual pairs (Supplementary method, Supplementary Table S5, and S6). With a 12-minute sequencing, a minimum of 11 loci were genotyped across the six individuals (2800 M, 4.27 Mbp), achieving a likelihood ratio surpassing 1.17e18 (Fig. 7c). According to the standards [31–33], a parent–child relationship is confirmable with a paternity index over 1e4. At 18 minutes, three parent–child relationships were successfully confirmed (Fig. 7d). It is important to note that a considerable portion of the sequencing data, including SNPs and sexual STRs amplicons, was not used in our analysis due to the ForenSeq system. This indicates that the duration for both identification tasks could be potentially reduced.

### Comparison of running time

NASTRA is developed using Python programming language and integrates the parasail library [34], which is a C-based pairwise alignment tool. When using NASTRA, users can accomplish parallel computing at the individual level by invoking the “xargs” shell command. For STRspy, the developers utilize the “parallel” shell command for parallelization at the locus level. To conduct the runtime comparisons, we allocated the same number of threads (eight) to both NASTRA and STRspy. The STR genotype calling for all sequencing data was conducted on a server equipped with a 64-Core/128-Thread AMD EPYC 7763 Processor (2.45GHz) and 256 GB of memory.

Table 1 presents a detailed runtime usage between NASTRA and STRspy. It reveals that NASTRA is ~60 times faster than STRspy when analyzing ForenSeq amplicon sequencing data. The PowerSeq data had more reads to process than the ForenSeq data because a smaller fraction of the sequencing throughput was invalidated. This resulted in a longer alignment runtime. Consequently, NASTRA demonstrates a significantly faster performance overall. However, the two tools are developed in different programming languages, making direct runtime comparisons somewhat subjective. Notably, our analysis shows that STRspy’s time-consuming alignment process, which includes individual executions of Minimap2 [35, 36] for each locus, contributes substantially to its longer runtime.

**Table 1:** Runtime comparison under the same thread count (eight threads)

Data source	Number of samples	Total reads	Total bases (Mb)	NASTRA (sec)	STRspy (sec)
ForenSeq + Guppy hac (24 hours)	16	5 722 882	1846.1	50.6	3605
	17	5 471 350	1805.6	62.1	3954
	23	4 024 251	1133.7	44.8	2690
	24	2 944 237	858.9	37.1	1664
PowerSeq + Guppy hac (24 hours)	24	4 398 698	1026.9	41.2	7481
	24	4 897 877	1281.1	48.4	6553
Eight human cell lines (24 hours)	8	5 013 171	1348.7	39.0	2422

## Conclusion and discussion

In this study, we introduced NASTRA, a novel computational approach designed for STR analysis. NASTRA is built on two primary modules: read clustering and repeat structure inference. To mitigate the influences of sequencing noises on genotyping calls, NASTRA employs read clustering. This approach identifies candidate alleles by discounting minor noises. Following this, the structure-aware algorithm reconstructs the repeat structures of alleles, drawing on known repeat motifs from fact sheets. This step converts the allele sequences into bracket sequences, thereby simplifying the identification of incomplete units, SNPs, or InDels. Compared to the sequential search method, the algorithm of recursive repeat searching in NASTRA has advantages in identifying sequence errors and handling alleles that differ from the known repeat structures (Supplementary Fig. S7). A key advantage of NASTRA over database-based methods is its independence from the allele reference database, enabling it to genotype STRs even when allele information is missing from the database. Moreover, we used this method on MiSeq FGx data during our benchmark construction, proving its applicability to NGS data as well.

To comprehensively evaluate NASTRA’s performance, we conducted a series of assessments involving various amplification kits and flow cells. These include analyses of ForenSeq data, PowerSeq data, and two practical case studies. Initially, we created a benchmark dataset comprising 84 DNA samples using the MiSeq FGx system, a widely-accepted technology for forensic applications. Then, we expanded the training and test sets to obtain the optimal thresholds for NASTRA. Further, we compared NASTRA with the database-based tools STRspy on ForenSeq data and PowerSeq data in terms of accuracy and speed. It is important to note that we did not include renowned repeat quantification tools like repeatHMM [25], STRique [26], and DeepRepeat [27] in our comparison. These tools are specifically designed for detecting repeat expansion disorders and may not provide the accuracy needed for forensic STR genotyping. Additionally, we conducted two case studies: cell line STR profiling and paternity testing. In summary, our results demonstrate that NASTRA excels in accuracy and speed, underscoring its potential as an effective method for real-world applications.

However, NASTRA has several limitations for further improvement. Firstly, the comparison was limited to only one database-based method, and the associated reference database is still under development. Consequently, this comparison might not offer a completely objective assessment but rather a representative illustration in terms of accuracy and runtime. Secondly, we briefly analyzed the Y-STR typing results using NASTRA, and the results showed that NASTRA has the potential to accurately type Y-STRs (Supplementary Figs S8 and S9). But further optimization for Y-STR is needed. Thirdly, there is a risk in NASTRA’s read clustering process that could lead to two alleles being misclassified as one, particularly in cases involving an SNP or a one-base gap between two similar alleles. Fourthly, our tests on ForenSeq and PowerSeq data revealed that amplicon length influences NASTRA’s performance. Moreover, the similarity in the flanking regions of STRs, including prefixes and suffixes, could result in incorrect trimming. Therefore, for accurate STR genotyping, NASTRA requires amplicons with extended flanking regions (>30 bp). Lastly, NASTRA’s effectiveness is contingent on the quality of base sequences input, as it relies heavily on the base calling model. Given that STRs are complex genomic regions, developing a robust basecalling model specifically for STRs is imperative.

**Key Points**

- NASTRA represents an innovative computational approach for accurate STR analysis and genotyping in nanopore sequencing data, employing a structure-aware algorithm that bypasses traditional alignment with a reference database.
- To assess the performance of NASTRA, we collected samples from 80 volunteers and 8 DNA standards and sequenced them using nanopore sequencing and Illumina MiSeq FGx system, which is the largest dataset to date in this research area.
- Our comprehensive evaluation process involved the use of two different amplification kits and the application of both R9.4.1 and R10 flow cells for sequencing. In a direct comparison with the database-based method, NASTRA exhibits superior performance in terms of speed and accuracy.
- Our case studies reveal NASTRA’s efficacy in ensuring cell line authentication, individual identification, and paternity testing within a minimal sequencing duration. These findings underscore NASTRA’s practical utility in real-world biomedical and forensic research scenarios.

## Supplementary Material

supplementary_bbae472

## Data Availability

NanoTime is available on GitHub (https://github.com/renzilin/NanoTime). NASTRA is under GPL v3.0 license and is publicly available on GitHub (https://github.com/renzilin/NASTRA). The data that support the findings of this study have been deposited into the CNGB Sequence Archive (CNSA) [37] of China National GeneBank DataBase (CNGBdb) [38] with accession number CNP0004956.
