# Supplementary material for: NASTRA: accurate analysis of short tandem repeat markers by nanopore sequencing with repeat-structure-aware algorithm
Source: Brief Bioinform. 2024 Sep 25;25(6):bbae472. doi: 10.1093/bib/bbae472 (PMC11424183; doi:10.1093/bib/bbae472)
Supplement: supplementary_bbae472 [file supplementary_bbae472.docx]

# Supplementary Method

## Dataset construction and Nanopore sequencing

**Sample collection and DNA extraction.** Genomic DNA from the newly collected samples was extracted using the PureLink Genomic DNA Kit (Invitrogen, USA). Before amplification, all DNA samples were quantified using the Qubit 3.0 Fluorometer (Invitrogen) and the Qubit dsDNA HS Assay Kit, following the manufacturer's instructions.

**Benchmarking data using MiSeq FGx system.** We amplified the newly collected DNA samples using the ForenSeq DNA Signature Prep Kit (DNA Primer Mix A). Concurrently, the 2800M sample was amplified as a positive control using DNA Primer Mix B, encompassing all STR loci present in the Mix A kit. To ensure ample amplicons for both Illumina MiSeq FGx and Nanopore sequencing, we used 50 ng of template DNA instead of the recommended 1 ng. The PCR amplifications were executed on the ProFlex™ 3x32-Well PCR System (Thermo Fisher). Subsequently, all purified amplified libraries were evenly split into two portions for sequencing on the MiSeq FGx system and Nanopore platform. The pooled libraries were prepared by combining equal volumes (2 μL) of each normalized library (10 nmol), which were then diluted to a final concentration of 4 nmol. For MiSeq FGx sequencing, 7 pmol of the pooled libraries were used. All experimental procedures were meticulously conducted in accordance with the manufacturer’s guidelines. Genotype analysis of the STR loci was conducted using the ForenSeq Universal Analysis Software, UAS (v1.3.6897; Verogen) with default parameters.

**Nanopore sequencing on ForenSeq amplicons.** Libraries were prepared with the Ligation Sequencing Kit (SQK-LSK109). The process involved processing 0.2 pmol of purified DNA per sample using the NEBNext Ultra II End repair/dA-tailing Module (E7546). Samples were then multiplexed utilizing both the Native Barcoding Expansion 1- 12 (PCR-free) and 13-24 (PCR-free) kits. Subsequently, adapters were ligated to the pooled libraries using the NEBNext Quick Ligation Module (E6056). A total of 0.05 pmol of these libraries were loaded on the R9.4.1 flow cells. The sequencing was carried out using MinKNOW software. Forty-six DNA samples, along with eight control DNA samples, underwent sequencing across three runs, each lasting over 24 hours. For the paternity testing, six samples were loaded on an R10.3 flow cell for sequencing.

**Nanopore sequencing for PowerSeq amplicons.** Following the manufacturer's guidelines, we randomly selected 46 DNA samples for amplification using the PowerSeq™ 46GY System (Promega, WI, USA). We utilized 1 ng of template DNA per sample and conducted PCR amplification over 29 cycles. Then, the PCR products were purified using the AmPure XP Beads (Beckman Coulter, Indianapolis, USA), and the DNA concentrations were quantified. The library preparation process mirrored the methodology previously described. The final step involved loading 0.05 pmol of the libraries on the R10.3 flow cells for sequencing, which was executed using MinKNOW software. In this phase, the 48 DNA samples and the 6 samples involved in the paternity testing case study underwent sequencing across 3 runs, each extending beyond a 24-hour duration.

**Capillary Electrophoresis for paternity testing.** For the paternity testing case study, we performed STR typing on a family of four individuals and two unrelated individuals, 2800M and 9948. The typing results were obtained by capillary electrophoresis and served as a benchmark. We used 1 μl of DNA template from each sample to amplify the targeted amplicons with the PowerPlex 21 kit (Promega) and the GeneAmp PCR System 9700 (Applied Biosystems). The PCR products were then separated using the 3130XL Series Genetic Analyzers (Applied Biosystems). Data analysis was conducted using the GeneMapper ID Software (version 3.2) (Applied Biosystems).

## Bioinformatic analysis for nanopore sequencing data

The basecalling process was performed using Guppy v6.3.4 with the high-accuracy model. Then, the reads were aligned to the human reference genome (assembly GRCh37, hg19) using Minimap2 v2.17-r941 [1],[2]. Finally, SAMtools v1.6 [3] were employed to convert SAM files into BAM format, which were then used as input for NASTRA. Down-sampled data were generated using NanoTime, which is available at https://github.com/renzilin/NanoTime. In this process, NanoTime utilized the timestamps of each read in the sequencing summary file to generate a series of FASTQ files with varying sequencing durations ranging from 1 to 24 hours (1, 2, 3, 4, 5, 6, 8, 10, 12, 16, 20, and 24 hours).

For the pairwise alignment used in NASTRA, we used parasail-python [4] (https://github.com/jeffdaily/parasail-python) to conduct a pairwise alignment with an affine-gap penalty in trimming and clustering steps. The parameters were set with a match score of 75, a mismatch penalty of 90, a gap opening penalty of 75, and a gap extension penalty of 10. As a result, this alignment configuration shows a tendency to prefer two consecutive gaps over a single mismatch. However, if the sequence has more than two continuous gaps, the alignment outcome becomes more inclined to favor a single mismatch.

## Individual identification and paternity index

In order to evaluate the effectiveness of individual identification, we used the Likelihood Ratio (LR) calculation method. This method is based on the allele frequency of each locus. For each locus, the allele frequency of the locus typing is obtained. If the two alleles of a site are the same, the likelihood ratio of the locus is the square of the allele frequency. If they are different, the likelihood ratio of the locus is 2 times the frequency product. The likelihood ratio of the locus is multiplied by the overall LR. Finally, 1/LR is used to determine whether the two samples come from the same individual.

In order to evaluate the validity of the parent-child relationship, we used the calculation method of the Paternity Index (PI). This method analyzes the genotype data of the father and the child based on the allele frequency of each locus. For each locus, extract the alleles and allele frequencies of the child and the father at the locus. According to the combination of alleles, calculate the parent-child relationship index at the locus: if the child and the father have the same alleles, the parent-child relationship index is the reciprocal of the allele frequency; if the child's alleles are the same and the father's alleles are different, or the child's alleles are different and the father's alleles are the same, the parent-child relationship index is half of the reciprocal of the corresponding allele frequency; if the child and the father have different alleles, it is necessary to consider the matching of the alleles to calculate the parent-child relationship index.

The code of LR and PI calculation has already been released on Github: <https://github.com/renzilin/NASTRA/tree/main/paternity_index_calculation>.

## ForenSeq Universal Analysis Software Terminology

According to the Universal Analysis Software Reference Guide (v2.0, <https://verogen.com/wp-content/uploads/2021/05/universal-analysis-software-v2-0-reference-guide-vd2019002-e.pdf>), the quality control is to filter the genotyping result that doesn’t meet the metric below:

**Allele Count.** The locus has more typed alleles than expected for a single-source sample of the indicated gender.

**Imbalanced.** The locus is not homozygous, not hemizygous, and falls below the intralocus balance setting.

**Analytical Threshold (AT).** The percentage defined in an analysis method that a read count must reach for the software to call a base or type an allele. Signal below the AT might be visible, but is not typed. The locus has expected allele signal below the AT and no alleles greater than or equal to the IT.

**Interpretation Threshold (IT).** The percentage defined in an analysis method that determines the read count that must be reached for the software to call a base or type an allele. Signal below the IT but greater than or equal to the AT can be manually typed. The locus has expected allele signal greater than or equal to the AT, but below IT.

**Stutter.** At least one sequence exceeds the stutter filter percentage at a stutter position of a possible parent allele.

## locus-specific adjustments

Since NASTRA relies on the motifs from STRbase for inferring repeated structures, the motifs it uses don't account for potential SNPs. Therefore, we have implemented adaptive adjustments in NASTRA for certain loci to ensure accurate genotyping. These adjustments focus on identifying SNPs in both the flanking regions and the repeat regions themselves. Below is a detailed description of these adjustments.

D13S317 is a simple TATC tetranucleotide repeat. In the 3’ flank adjacent to the repeat region is AATC AATC CTCA. Due to polymorphisms [5], the sequence can change to TATC AATC CTCA or TATC TATC CTCA, potentially leading to misinterpretation of the TATC sequence as part of the repeat. To address this, NASTRA modifies its genotyping calls, taking into consideration the number of TATC sequences present in this adjacent area.

D5S818 is a simple ATCT tetranucleotide repeat. In the 5’ flank adjacent to the repeat region is CTCT. An SNP might alter the sequence from CTCT to ATCT [6], potentially leading to the misidentification of ATCT as part of the repeat. To accurately address this, NASTRA adjusts its genotyping calls by specifically identifying and accounting for the CTCT sequence.

D20S482 is a simple AGAT tetranucleotide repeat. In the 3’ flank close to the repeat region, the sequence contains AGCT. A possible SNP could change this to AGAT, which might then be mistakenly identified as part of the repeat sequence. To counter this issue, NASTRA modifies its genotyping calls by specifically recognizing and accounting for the AGCT sequence.

D2S1338 is characterized by a compound tetranucleotide repeat with a structure of [GGAA]n [GGAC]m [GGAA]x [GGCA]y. Polymorphisms can occur in [GGAA]n, leading to a transformation of GGAA into GAAA (3). To address this, NASTRA revises its genotyping calls by considering the length of nucleotides, including those not typically counted in the allele.

D2S441 contains a simple TCTA repeat. There are compound motifs consisting of a varying number of TCTA repeats, followed by a single tetranucleotide of either TCAA, TTTA, or TCTG, and concluding with more TCTA repeats. The total count for these compound motifs varies from 10 to 17 repeats, in the format [TCTA]n [TNNN] [TCTA]n [7,8]. NASTRA modifies the genotyping calls by the length of the TNNN.

D4S2408 is characterized by a simple ATCT repeat, with the motif structured as [ATCT]n. However, due to the presence of SNPs within the motif, the repeat structure can vary to [ATCT]m GTCT [ATCT]n. In response to this variation, NASTRA adapts its genotyping calls by considering the length of nucleotides that are not typically included in the allele count.

D7S820 features a simple TCTA repeat with the motif represented as [TCTA]n. However, the presence of SNPs within this motif can alter the repeat structure to [TCTA]m CCTA [TCTA]n [9]. In response to these variations, NASTRA adjusts its genotyping calls by considering the length of nucleotides that are typically not included in the allele count.

D18S51 is characterized by a simple AGAA repeat, with the motif typically structured as [AGAA]n. However, the presence of SNPs within this motif can lead to a variation in the repeat sequence, resulting in a structure like [AGAA]m AGCA [AGAA]n. To address this, NASTRA revises its genotyping calls, taking into account the length of nucleotides that are not conventionally included in the allele count.

D9S1122 features a simple TAGA repeat. The typical motif structure is [AGAA]n, but we have identified SNPs within this motif that can alter the repeat to [TAGA]1 TCGA [TAGA]n. Consequently, NASTRA modifies its genotyping calls by factoring in the length of nucleotides that aren't typically counted in the allele.

The vWA locus is known for its compound tetranucleotide repeat, with a possible repeat structure of [TAGA]x [CAGA]y [TAGA]z. Polymorphisms within [TAGA]n can lead to a conversion of TAGA into TGGA [10]. In response to this, NASTRA adapts its genotyping calls by considering the length of nucleotides that are typically not included in the allele count.

**References**

1. Li H. Minimap2: pairwise alignment for nucleotide sequences. Bioinformatics 2018; 34:3094–3100

2. Li H. New strategies to improve minimap2 alignment accuracy. Bioinformatics 2021; 37:4572–4574

3. Danecek P, Bonfield JK, Liddle J, et al. Twelve years of SAMtools and BCFtools. GigaScience 2021; 10:giab008

4. Daily J. Parasail: SIMD C library for global, semi-global, and local pairwise sequence alignments. BMC Bioinformatics 2016; 17:81

5. Wang L, Zhao X-C, Ye J, et al. Construction of a library of cloned short tandem repeat (STR) alleles as universal templates for allelic ladder preparation. Forensic Science International: Genetics 2014; 12:136–143

6. Allor C, Einum DD, Scarpetta M. Identification and characterization of variant alleles at CODIS STR loci. J Forensic Sci 2005; 50:1128–1133

7. Gettings KB, Kiesler KM, Faith SA, et al. Sequence variation of 22 autosomal STR loci detected by next generation sequencing. Forensic Sci Int Genet 2016; 21:15–21

8. Phillips C, Fernandez-Formoso L, Garcia-Magariños M, et al. Analysis of global variability in 15 established and 5 new European Standard Set (ESS) STRs using the CEPH human genome diversity panel. Forensic Sci Int Genet 2011; 5:155–169

9. Odriozola A, Aznar JM, Valverde L, et al. SNPSTR rs59186128_D7S820 polymorphism distribution in European Caucasoid, Hispanic, and Afro-American populations. Int J Legal Med 2009; 123:527–533

10. Gettings KB, Aponte RA, Vallone PM, et al. STR allele sequence variation: Current knowledge and future issues. Forensic Sci Int Genet 2015; 18:118–130

**Supplementary Tables**

**Supplementary Table S1.** The performance summary of NASTRA on the expanded test data.

|  | locus | FGx QC | Nastra QC | Calling Ratio | Accuracy |
| --- | --- | --- | --- | --- | --- |
| 1 | CSF1PO | 12 | 110 | 0.765 | 1 |
| 2 | D13S317 | 12 | 14 | 0.97 | 1 |
| 3 | D17S1301 | 12 | 51 | 0.891 | 1 |
| 4 | D20S482 | 12 | 3 | 0.994 | 1 |
| 5 | D21S11 | 24 | 95 | 0.792 | 1 |
| 6 | D3S1358 | 0 | 3 | 0.994 | 1 |
| 7 | D4S2408 | 12 | 22 | 0.953 | 1 |
| 8 | D5S818 | 48 | 120 | 0.722 | 1 |
| 9 | D8S1179 | 0 | 15 | 0.969 | 1 |
| 10 | D9S1122 | 0 | 12 | 0.975 | 1 |
| 11 | TH01 | 12 | 0 | 1 | 1 |
| 12 | TPOX | 12 | 95 | 0.797 | 1 |
| 13 | vWA | 24 | 143 | 0.686 | 1 |
| 14 | D10S1248 | 0 | 20 | 0.958 | 0.998 |
| 15 | D16S539 | 0 | 18 | 0.963 | 0.996 |
| 16 | D19S433 | 12 | 34 | 0.927 | 0.991 |
| 17 | D12S391 | 12 | 33 | 0.929 | 0.984 |
| 18 | D6S1043 | 24 | 33 | 0.928 | 0.976 |
| 19 | D2S441 | 24 | 0 | 1 | 0.974 |
| 20 | D1S1656 | 12 | 120 | 0.744 | 0.951 |
| 21 | D7S820 | 96 | 37 | 0.904 | 0.934 |
| 22 | PentaD | 48 | 419 | 0.03 | 0.923 |
| 23 | D2S1338 | 0 | 30 | 0.938 | 0.911 |
| 24 | D22S1045 | 360 | 19 | 0.842 | 0.901 |
| 25 | PentaE | 156 | 263 | 0.188 | 0.885 |
| 26 | D18S51 | 0 | 54 | 0.887 | 0.723 |
| 27 | FGA | 12 | 59 | 0.874 | 0.675 |

**Supplementary Table S2.** The accuracy comparison between NASTRA and STRspy on the ForenSeq data.

|  | locus | NASTRA | STRspy |
| --- | --- | --- | --- |
| 1 | D10S1248 | 1 | 1 |
| 2 | D12S391 | 1 | 1 |
| 3 | D16S539 | 1 | 1 |
| 4 | D17S1301 | 1 | 1 |
| 5 | D20S482 | 1 | 1 |
| 6 | D3S1358 | 1 | 1 |
| 7 | D5S818 | 1 | 1 |
| 8 | D8S1179 | 1 | 1 |
| 9 | D9S1122 | 1 | 1 |
| 10 | TH01 | 1 | 1 |
| 11 | TPOX | 1 | 1 |
| 12 | CSF1PO | 1 | 0.975 |
| 13 | D4S2408 | 1 | 0.974 |
| 14 | D21S11 | 1 | 0.921 |
| 15 | D19S433 | 1 | 0.846 |
| 16 | D13S317 | 1 | 0.7 |
| 17 | D22S1045 | 1 | 0.7 |
| 18 | vWA | 1 | 0.684 |
| 19 | D2S441 | 0.974 | 1 |
| 20 | D1S1656 | 0.973 | 0.95 |
| 21 | D6S1043 | 0.972 | 0.974 |
| 22 | D7S820 | 0.938 | 0.938 |
| 23 | D2S1338 | 0.925 | 1 |
| 24 | PentaE | 0.909 | 0.885 |
| 25 | PentaD | 0.875 | 1 |
| 26 | D18S51 | 0.8 | 0.825 |
| 27 | FGA | 0.784 | 0.789 |

**Supplementary Table S3.** The accuracy comparison between NASTRA and STRspy on PowerSeq data.

|  | locus | NASTRA | STRspy |
| --- | --- | --- | --- |
| 1 | CSF1PO | 1 | 1 |
| 2 | D10S1248 | 1 | 1 |
| 3 | D12S391 | 1 | 1 |
| 4 | D16S539 | 1 | 1 |
| 5 | D22S1045 | 1 | 1 |
| 6 | D3S1358 | 1 | 1 |
| 7 | D5S818 | 1 | 1 |
| 8 | D8S1179 | 1 | 1 |
| 9 | TPOX | 1 | 1 |
| 10 | D19S433 | 1 | 0.978 |
| 11 | D1S1656 | 1 | 0.978 |
| 12 | D2S1338 | 1 | 0.957 |
| 13 | D21S11 | 1 | 0.935 |
| 14 | D13S317 | 1 | 0.787 |
| 15 | vWA | 1 | 0.638 |
| 16 | D18S51 | 1 | 0.13 |
| 17 | D2S441 | 0.979 | 1 |
| 18 | TH01 | 0.979 | 1 |
| 19 | D7S820 | 0.952 | 0.929 |
| 20 | PentaE | 0.919 | 0.921 |
| 21 | PentaD | 0.8 | 1 |
| 22 | FGA | 0 | 0.761 |

**Supplementary Table S4.** Throughput per sample across different sequencing durations in the paternity test. Notably, the throughput values are calculated using the entire sequencing data from the ForenSeq amplicons, even though some parts of this data are not utilized by NASTRA.

|  |  |  | **Samples** | | | | |
| --- | --- | --- | --- | --- | --- | --- | --- |
|  | **throughput (Mbp)** | **2800M** | **9948** | **C** | **F** | **M** | **GM** |
| **Sequencing durations** | 6 min | 1.90 | 2.52 | 2.35 | 2.74 | 1.84 | 1.96 |
|  | 12 min | 4.27 | 5.59 | 5.14 | 6.12 | 4.02 | 4.43 |
|  | 18 min | 6.58 | 8.59 | 7.89 | 9.42 | 6.16 | 6.89 |
|  | 24 min | 8.71 | 11.56 | 10.53 | 12.65 | 8.16 | 9.28 |
|  | 30 min | 10.80 | 14.38 | 13.04 | 15.78 | 10.08 | 11.48 |
|  | 1h | 20.64 | 26.95 | 24.41 | 29.25 | 18.38 | 21.26 |
|  | 2h | 34.83 | 46.76 | 43.43 | 50.26 | 31.15 | 36.48 |
|  | 3h | 47.64 | 64.69 | 59.44 | 69.42 | 42.50 | 50.38 |
|  | 4h | 58.70 | 80.04 | 73.01 | 85.84 | 52.29 | 62.18 |
|  | 5h | 64.94 | 88.35 | 81.54 | 95.50 | 58.48 | 69.67 |

**Supplementary Table S5.** The likelihood ratio for Individual identification.

| **individual** | **6min** | **12min** | **18min** | **24min** | **30min** | **1h** | **2h** | **3h** | **4h** | **5h** |
| --- | --- | --- | --- | --- | --- | --- | --- | --- | --- | --- |
| **2800M** | 2.22E+03 | 1.17E+18 | 6.25E+24 | 3.52E+28 | 3.69E+32 | 3.69E+32 | 3.69E+32 | 3.69E+32 | 3.69E+32 | 3.69E+32 |
| **9948** | 1.46E+11 | 7.57E+21 | 1.17E+21 | 3.28E+24 | 2.03E+27 | 1.81E+28 | 1.81E+28 | 1.81E+28 | 1.81E+28 | 1.81E+28 |
| **C** | 2.79E+15 | 1.79E+24 | 2.99E+26 | 5.84E+27 | 1.32E+31 | 3.76E+32 | 3.76E+32 | 3.76E+32 | 3.76E+32 | 3.76E+32 |
| **F** | 3.88E+10 | 4.03E+19 | 3.08E+23 | 1.60E+28 | 1.60E+28 | 9.04E+28 | 9.04E+28 | 9.04E+28 | 9.04E+28 | 9.04E+28 |
| **GM** | 4.69E+07 | 5.00E+18 | 4.02E+19 | 1.92E+25 | 1.30E+30 | 1.30E+30 | 1.30E+30 | 1.30E+30 | 1.30E+30 | 1.30E+30 |
| **M** | 2.72E+10 | 1.35E+15 | 4.11E+19 | 4.99E+25 | 6.87E+27 | 1.03E+31 | 1.03E+31 | 1.03E+31 | 1.03E+31 | 1.03E+31 |

**Supplementary Table S6.** The Paternity Index for paternity testing.

| **Individual 1** | **Individual 2** | **6min** | **12min** | **18min** | **24min** | **30min** | **1h** | **2h** | **3h** | **4h** | **5h** |
| --- | --- | --- | --- | --- | --- | --- | --- | --- | --- | --- | --- |
| **M** | **F** | 0 | 0 | 0 | 0 | 0 | 0 | 0 | 0 | 0 | 0 |
| **M** | **C** | 24.80 | 9.14E+02 | 2.09E+04 | 7.02E+05 | 7.02E+05 | 1.75E+08 | 1.75E+08 | 1.75E+08 | 1.75E+08 | 1.75E+08 |
| **GM** | **M** | 179.92 | 1.81E+04 | 1.02E+05 | 3.66E+06 | 1.91E+07 | 1.49E+09 | 1.49E+09 | 1.49E+09 | 1.49E+09 | 1.49E+09 |
| **GM** | **F** | 0 | 0 | 0 | 0 | 0 | 0 | 0 | 0 | 0 | 0 |
| **GM** | **C** | 0 | 0 | 0 | 0 | 0 | 0 | 0 | 0 | 0 | 0 |
| **F** | **C** | 1.96E+03 | 9.95E+04 | 2.04E+07 | 9.04E+08 | 3.36E+09 | 3.64E+09 | 3.64E+09 | 3.64E+09 | 3.64E+09 | 3.64E+09 |
| **2800M** | **M** | 2.54 | 0 | 0 | 0 | 0 | 0 | 0 | 0 | 0 | 0 |
| **2800M** | **GM** | 2.54 | 0 | 0 | 0 | 0 | 0 | 0 | 0 | 0 | 0 |
| **2800M** | **F** | 0 | 0 | 0 | 0 | 0 | 0 | 0 | 0 | 0 | 0 |
| **2800M** | **C** | 2.54 | 0 | 0 | 0 | 0 | 0 | 0 | 0 | 0 | 0 |
| **9948** | **M** | 0 | 0 | 0 | 0 | 0 | 0 | 0 | 0 | 0 | 0 |
| **9948** | **GM** | 0 | 0 | 0 | 0 | 0 | 0 | 0 | 0 | 0 | 0 |
| **9948** | **F** | 0 | 0 | 0 | 0 | 0 | 0 | 0 | 0 | 0 | 0 |
| **9948** | **C** | 0 | 0 | 0 | 0 | 0 | 0 | 0 | 0 | 0 | 0 |
| **9948** | **2800M** | 8.73 | 0 | 0 | 0 | 0 | 0 | 0 | 0 | 0 | 0 |

**Supplementary Table S7.** Sample information in ForenSeq dataset, PowerSeq dataset, and DNA standards.

| **ForenSeq dataset** | | | | | |
| --- | --- | --- | --- | --- | --- |
| **pool** | **barcode** | **sample** | **pool** | **barcode** | **sample** |
| 1 | barcode01 | M1 | 3 | barcode09 | F24 |
| 1 | barcode02 | M2 | 3 | barcode10 | F25 |
| 1 | barcode03 | M3 | 3 | barcode11 | F26 |
| 1 | barcode04 | M4 | 3 | barcode12 | F27 |
| 1 | barcode05 | M5 | 3 | barcode13 | F28 |
| 1 | barcode06 | M6 | 3 | barcode14 | F29 |
| 1 | barcode07 | M7 | 3 | barcode15 | F30 |
| 1 | barcode08 | M8 | 3 | barcode16 | F31 |
| 1 | barcode09 | M9 | 3 | barcode17 | F32 |
| 1 | barcode10 | M10 | 3 | barcode18 | F33 |
| 1 | barcode11 | M11 | 3 | barcode19 | F34 |
| 1 | barcode12 | M12 | 3 | barcode20 | F35 |
| 1 | barcode13 | M13 | 3 | barcode21 | F36 |
| 1 | barcode14 | M14 | 3 | barcode22 | F37 |
| 1 | barcode15 | M15 | 3 | barcode23 | 2800M (4) |
| 1 | barcode16 | 2800M (1) | 3 | barcode24 | NTC |
| 2 | barcode01 | F9 | 4 | barcode01 | F38 |
| 2 | barcode03 | F10 | 4 | barcode02 | M16 |
| 2 | barcode04 | F11 | 4 | barcode03 | M17 |
| 2 | barcode05 | F12 | 4 | barcode04 | M18 |
| 2 | barcode06 | F13 | 4 | barcode05 | M19 |
| 2 | barcode07 | F14 | 4 | barcode06 | M20 |
| 2 | barcode08 | F15 | 4 | barcode07 | M21 |
| 2 | barcode09 | 2800M (2) | 4 | barcode08 | M22 |
| 2 | barcode10 | 2800M (3) | 4 | barcode09 | M23 |
| 2 | barcode17 | F1 | 4 | barcode10 | M24 |
| 2 | barcode18 | F2 | 4 | barcode11 | M25 |
| 2 | barcode19 | F3 | 4 | barcode12 | M26 |
| 2 | barcode20 | F4 | 4 | barcode13 | M37 |
| 2 | barcode21 | F5 | 4 | barcode14 | M38 |
| 2 | barcode22 | F6 | 4 | barcode15 | M27 |
| 2 | barcode23 | F7 | 4 | barcode16 | M28 |
| 2 | barcode24 | F8 | 4 | barcode17 | M29 |
| 3 | barcode01 | F16 | 4 | barcode18 | M32 |
| 3 | barcode02 | F17 | 4 | barcode19 | M33 |
| 3 | barcode03 | F18 | 4 | barcode20 | M34 |
| 3 | barcode04 | F19 | 4 | barcode21 | M35 |
| 3 | barcode05 | F20 | 4 | barcode22 | M36 |
| 3 | barcode06 | F21 | 4 | barcode23 | M30 |
| 3 | barcode07 | F22 | 4 | barcode24 | M31 |
| 3 | barcode08 | F23 |  |  |  |
| **PowerSeq dataset** | | | | | |
| **pool** | **barcode** | **Sample** | **pool** | **barcode** | **sample** |
| 1 | barcode01 | F1 | 2 | barcode01 | M5 |
| 1 | barcode02 | F2 | 2 | barcode02 | M6 |
| 1 | barcode03 | F3 | 2 | barcode03 | M8 |
| 1 | barcode04 | F4 | 2 | barcode04 | M10 |
| 1 | barcode05 | F5 | 2 | barcode05 | M11 |
| 1 | barcode06 | F6 | 2 | barcode06 | M12 |
| 1 | barcode07 | F7 | 2 | barcode07 | M13 |
| 1 | barcode08 | F8 | 2 | barcode08 | M14 |
| 1 | barcode09 | F9 | 2 | barcode09 | M15 |
| 1 | barcode10 | F10 | 2 | barcode10 | M21 |
| 1 | barcode11 | F12 | 2 | barcode11 | M23 |
| 1 | barcode12 | F13 | 2 | barcode12 | M24 |
| 1 | barcode13 | F14 | 2 | barcode13 | M26 |
| 1 | barcode14 | F17 | 2 | barcode14 | M27 |
| 1 | barcode15 | F18 | 2 | barcode15 | M28 |
| 1 | barcode16 | F24 | 2 | barcode16 | M30 |
| 1 | barcode17 | F25 | 2 | barcode17 | 2800M |
| 1 | barcode18 | F26 | 2 | barcode18 | 9947A |
| 1 | barcode19 | F27 | 2 | barcode19 | 9948 |
| 1 | barcode20 | F30 | 2 | barcode20 | NA12878 |
| 1 | barcode21 | M1 | 2 | barcode21 | NA24149 |
| 1 | barcode22 | M2 | 2 | barcode22 | NA24143 |
| 1 | barcode23 | NA24694 | 2 | barcode23 | M3 |
| 1 | barcode24 | M4 | 2 | barcode24 | NA24695 |
| **DNA standards** | | | | | |
| **pool** | **barcode** | **sample** | **pool** | **barcode** | **sample** |
| 5 | barcode01 | NA12878 | 5 | barcode05 | NA24695 |
| 5 | barcode02 | NA24143 | 5 | barcode06 | 9947A |
| 5 | barcode03 | NA24149 | 5 | barcode07 | 9948 |
| 5 | barcode04 | NA24694 | 5 | barcode08 | 2800M |

**Supplementary Figures**


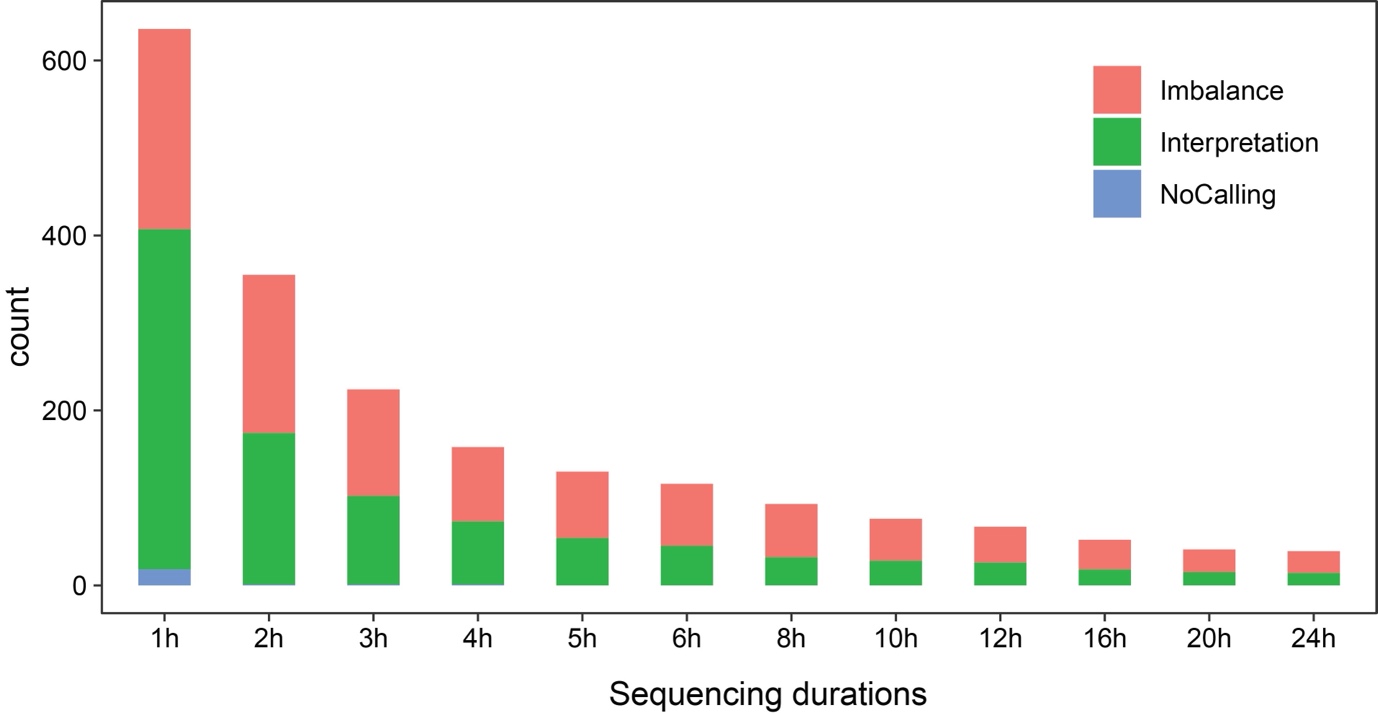


**Supplementary Figure S1.** The number of failed genotyping results with various sequencing durations in downsampled data. Blue represents NoCalling, which indicates that the number of reads spanning the corresponding region is fewer than 10. Red represents for Imbalance, indicating the SN of the minor allele is less than 25. Green represents Interpretation, indicating the SN of the major allele is less than 25.

**Supplementary Figure S2.** The calling ratio of NASTRA and STRspy in the ForenSeq data. For STRspy, we built the allele reference database for D17S1301, D20S482, D4S2408, D9S1122, and D6S1043 from the STRBase.

**Supplementary Figure S3.** The calling ratio of NASTRA and STRspy in the PowerSeq data.


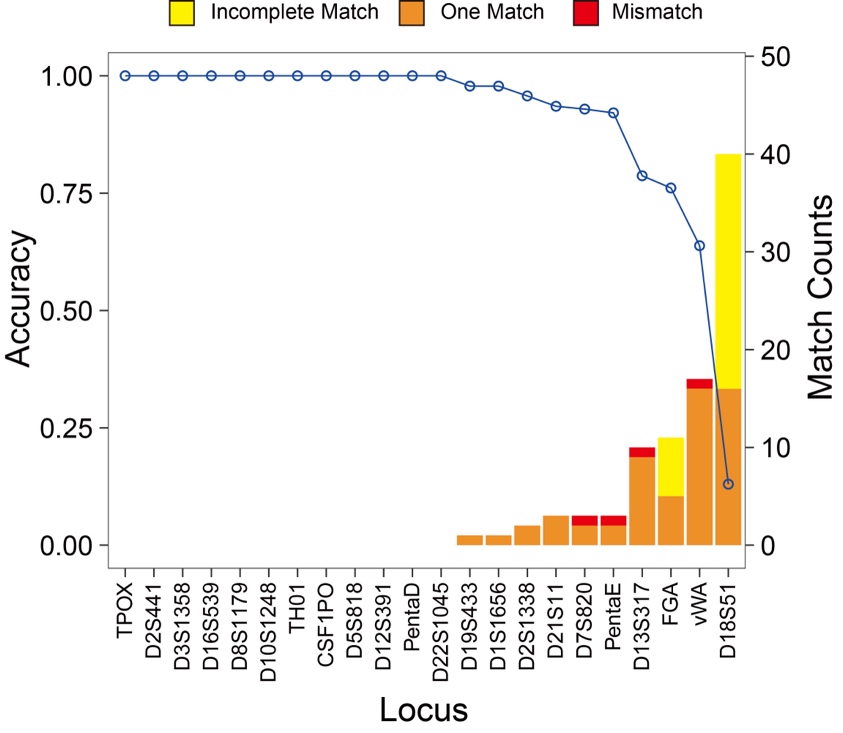


**Supplementary Figure S4.** The genotyping accuracy of STRspy in the PowerSeq data.


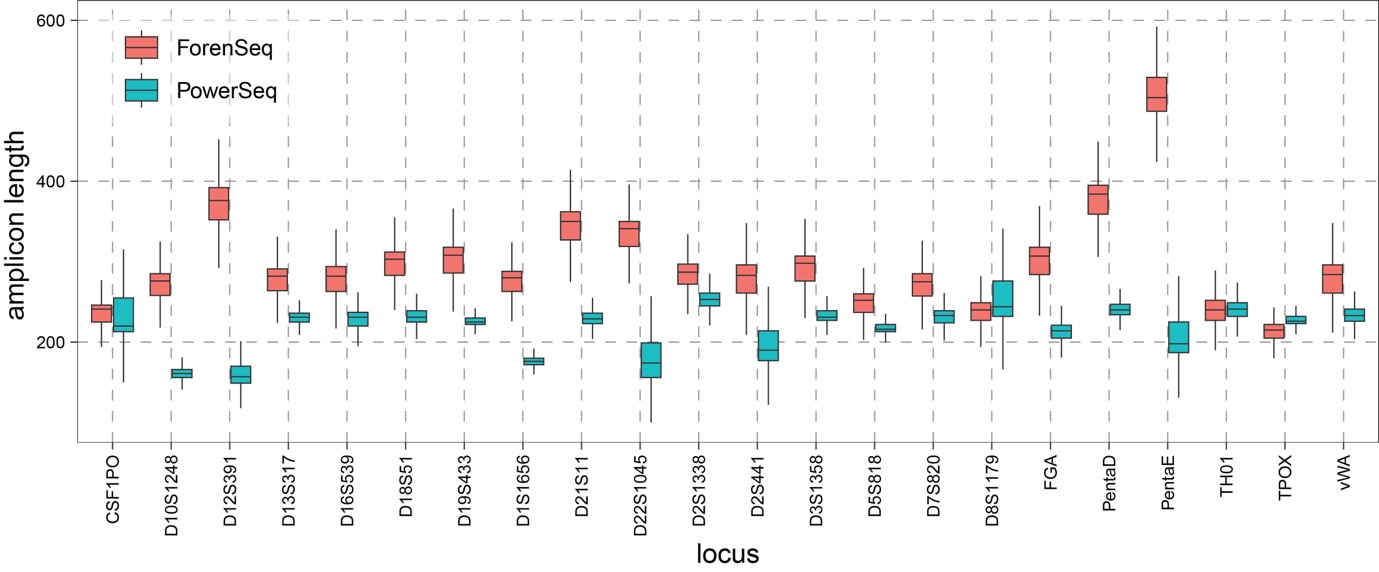


**Supplementary Figure S5.** The length distribution of amplicons in (a) the ForenSeq data and (b) the PowerSeq data. Overall, the amplicon length of the ForenSeq data is slightly longer than that of the PowerSeq data.

**Supplementary Figure S6.** The genotype distribution of the ForenSeq data.


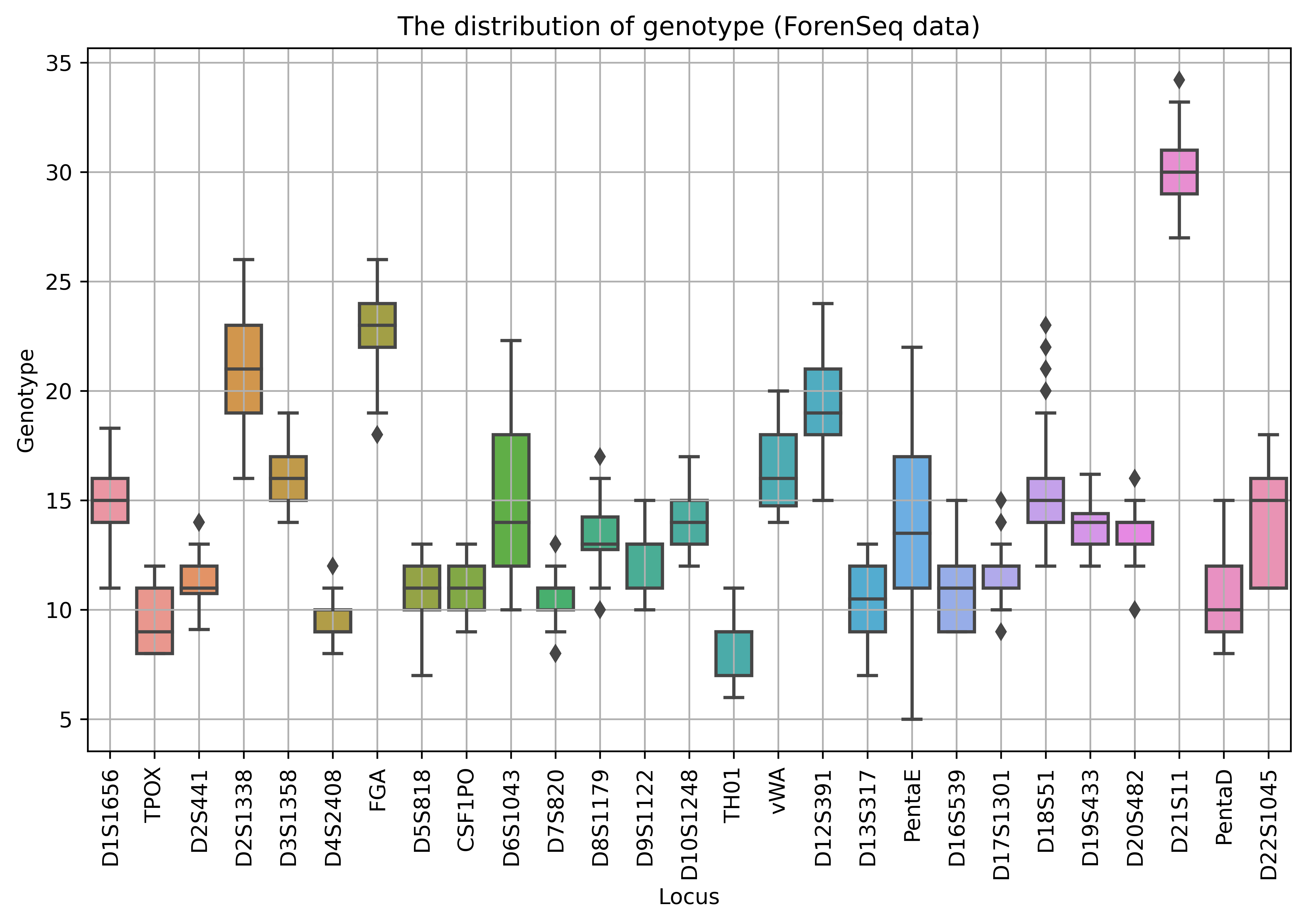

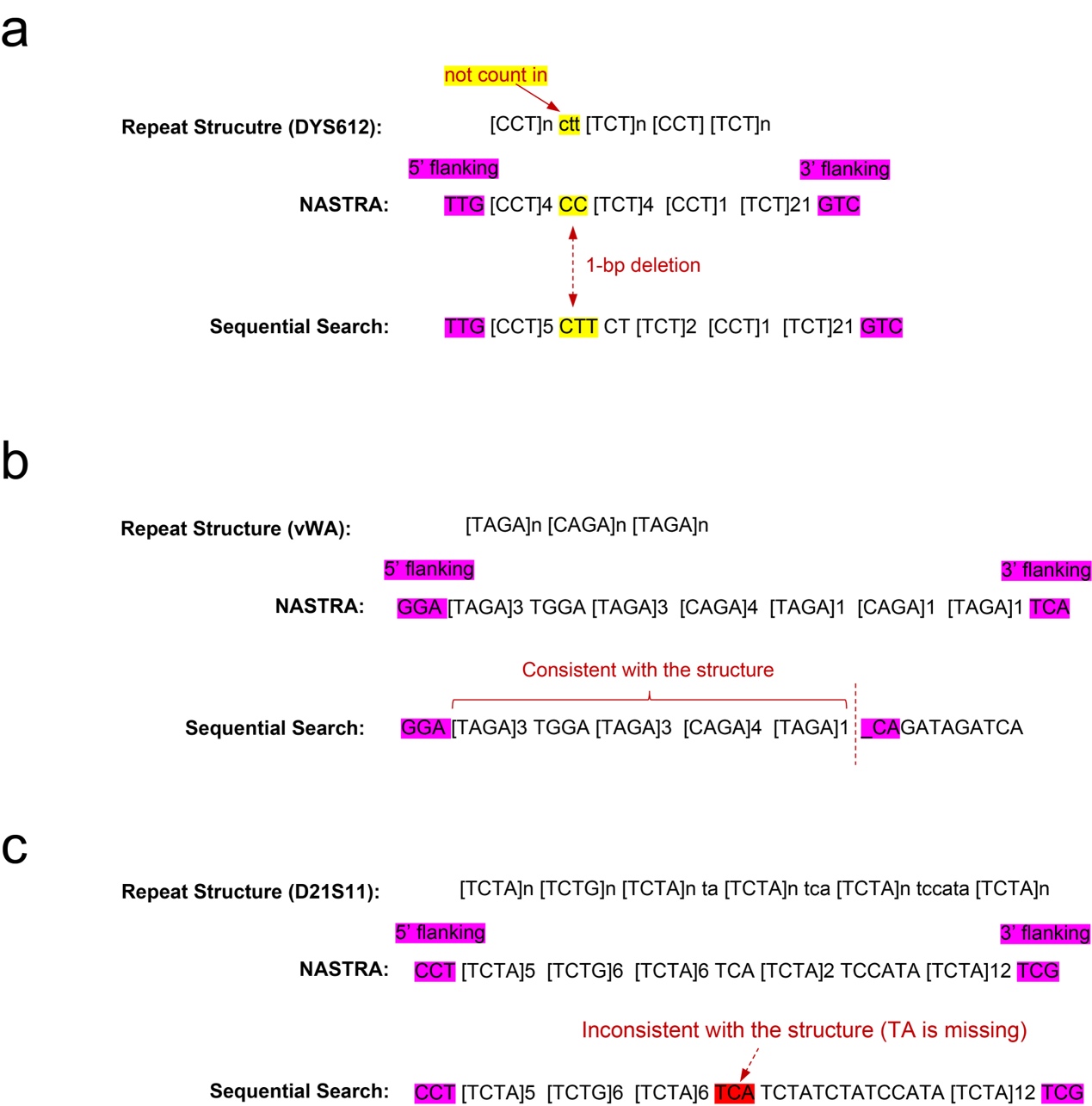


**Supplementary Figure S7.** Examples of comparing NASTRA’s repeat structure inference and sequence search method.


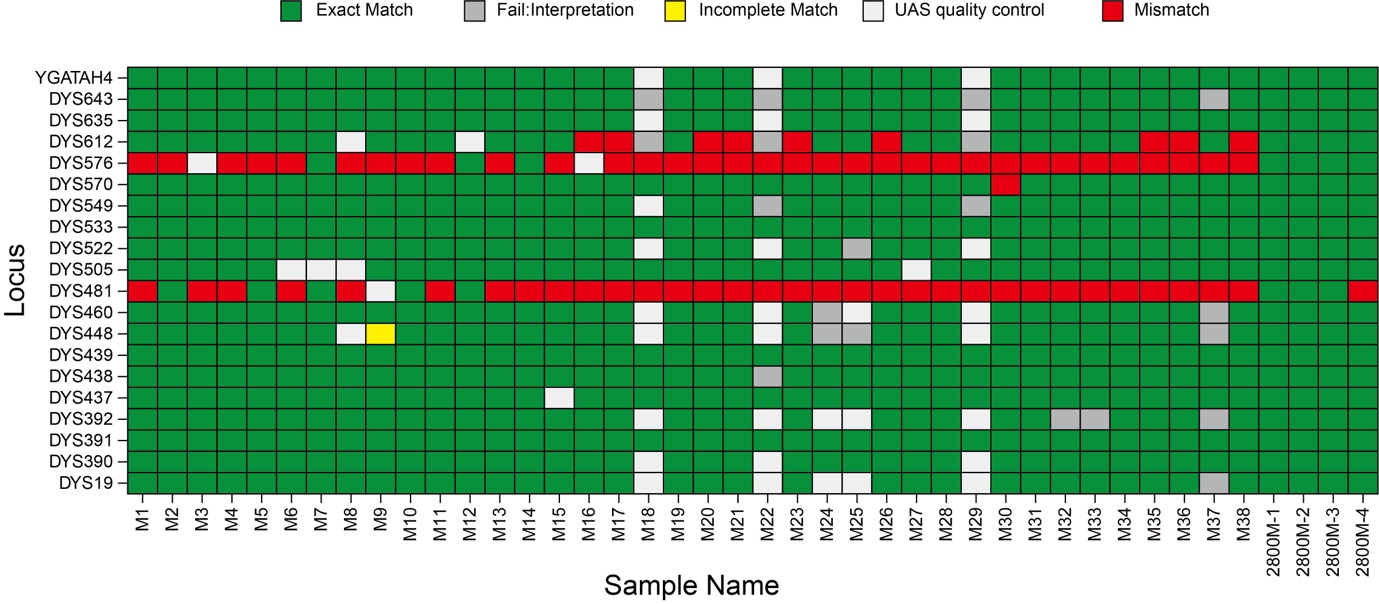


**Supplementary Figure S8.** The genotyping accuracy of NASTRA in the ForenSeq data.


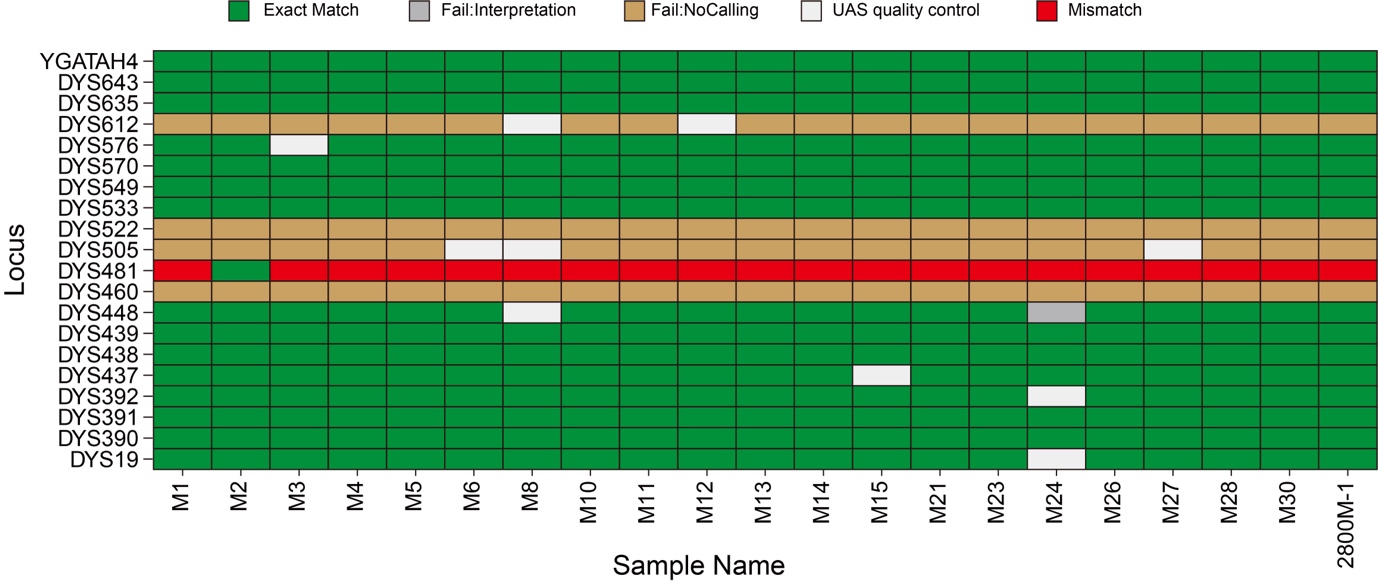


**Supplementary Figure S9.** The genotyping accuracy of NASTRA in the PowerSeq data
